# The Effect of Palmitoylethanolamide on Pain Intensity, Central and Peripheral Sensitization, and Pain Modulation in Healthy Volunteers—A Randomized, Double-Blinded, Placebo-Controlled Crossover Trial

**DOI:** 10.3390/nu14194084

**Published:** 2022-10-01

**Authors:** Kordula Lang-Illievich, Christoph Klivinyi, Gudrun Rumpold-Seitlinger, Christian Dorn, Helmar Bornemann-Cimenti

**Affiliations:** Department of Anaesthesiology and Intensive Care Medicine, Medical University of Graz, 8036 Graz, Austria

**Keywords:** palmitoylethanolamide, pain, central sensitization, peripheral sensitization, conditioned pain modulation, allodynia, hyperalgesia, wind-up

## Abstract

Palmitoylethanolamide (PEA) is marketed as a “dietary food for special medical purposes”. Its broad-spectrum analgesic, anti-inflammatory, and neuroprotective effects make PEA an interesting substance in pain management. However, the underlying analgetic mechanisms have not yet been investigated in humans. The aim of our study is to provide a deeper understanding of the involved mechanisms, which is essential for differentiating therapeutic approaches and the establishment of mechanism-based therapeutic approaches. In this randomized, placebo-controlled, double-blinded crossover trial, 14 healthy volunteers were included. PEA (3 × 400 mg per day) or placebo were taken for 4 weeks. Our study investigated the mode of action of PEA using an established pain model, “Repetitive phasic heat application”, which is well-suited to investigate analgesic and anti-hyperalgesic effects in healthy volunteers. Parameters for peripheral and central sensitization as well as for pain modulation were assessed. Repetitive heat pain was significantly decreased, and the cold pain tolerance was significantly prolonged after the PEA treatment. The pressure pain tolerance and the conditioned pain modulation were increased after the PEA treatment. The wind-up ratio and the average distance of allodynia were significantly decreased after the PEA treatment. The heat pain tolerance was significantly higher after the PEA treatment. The present study has demonstrated that PEA has clinically relevant analgesic properties, acting on both peripheral and central mechanisms as well as in pain modulation.

## 1. Introduction

The pharmacological treatment of acute and chronic pain is still a challenging problem. The efficacy of currently available medications is unsatisfactory [1]. Frequently used analgetic drugs, e.g., NSAIDs and opioids, are notorious for considerable safety risks [2,3,4,5]. The outlook of analgesic drugs in the near future does not appear promising because of unsatisfactory advancement in the development of novel therapeutic agents [1]. Keeping these facts in mind, known substances that are potentially efficacious in the treatment of acute and/or chronic pain must be investigated more rigorously.

Palmitoylethanolamide (PEA) is marketed as a “dietary food for special medical purposes”. As an endogenous fatty acid amide from the group of N-acetylethanolamides, it is a cannabimimetic compound. Different modes of action have been identified in preclinical studies. PEA does not bind to the classical cannabinoid receptors, but it indirectly stimulates endocannabinoids. It inhibits the enzyme that catalyzes the degradation of the endocannabinoid anandamide (AEA), which leads to higher levels of AEA in tissues, thus enhancing analgesic action [6]. PEA has shown an agonistic effect after binding to the peroxisome proliferator-activated receptor-α (PPAR-α) and transient receptor potential vanilloid type 1 (TRPV1) [7,8] as well as by binding to “CB2-like” receptors [9]. PEA also plays an important role in suppressing inflammation by inhibiting mast cell activation and by reducing the activity of proinflammatory enzymes such as eNOS, iNOS, and COX [7,10]. Overall, PEA makes a substantial contribution to regulating neurogenic inflammation [11].

The broad-spectrum analgesic, anti-inflammatory, and neuroprotective effects make PEA an interesting substance in pain management [12]. A recent meta-analysis of studies in which PEA was used as a treatment for neuropathic or chronic pain proved its clinical efficacy [13]. However, the underlying analgetic mechanisms have not yet been investigated in humans. 

Regarding the modes of action of analgesic drugs, three pivotal mechanisms can be differentiated: peripheral sensitization, central sensitization, and pain modulation. Peripheral sensitization occurs at the level of nociceptors. It causes primary hyperalgesia, which is due to excessive stimulation and a subsequent lowering of the threshold for nociception in the peripheral nervous system. It is clinically evident that primary hyperalgesia corresponds to a lowered heat pain threshold (HPT) [14,15].

In contrast, central sensitization occurs at the spinal and supraspinal levels. Clinically, it is characterized by secondary hyperalgesia and can be quantified by mechanical parameters such as the mechanical pain sensitivity (MPS), the area of allodynia, the pressure pain threshold, and cold pain tolerance [16].

Changes in nociceptive processing are reflected by dynamic pain measurements, i.e., wind-up and conditioned pain modulation (CPM) [17]. Wind-up is a frequency-dependent increase in the excitability of spinal cord neurons [18], whereas CPM captures endogenous pain modulation. Both are regarded as biomarkers of central pain mechanisms [19].

“Repetitive phasic heat application” is a validated method for inducing short-term peripheral and central sensitization. As a noninvasive human pain model, it is therefore well-suited to investigate the analgesic and anti-hyperalgesic effects of pharmaceuticals [20].

Our study investigated the mode of action of PEA using an established pain model in healthy volunteers, providing a deeper understanding of the involved mechanisms, which is essential for differentiating therapeutic approaches and the establishment of mechanism-based therapeutic approaches [16].

## 2. Materials and Methods

### 2.1. Trial Design and Setting

This study was designed as a randomized, placebo-controlled crossover trial conducted on healthy volunteers. After the ethics committee’s approval (review board number: 32-002 ex 19/20) and the registration of the protocol (www.clinicaltrials.gov, NCT04662827, accessed on 12 August 2022), this study was conducted at the Medical University of Graz, Austria, in accordance with all relevant regulations. The article was prepared by adhering to the Consort statement [21].

### 2.2. Trial Participants

Healthy adult volunteers (age ≥ 18) were recruited as participants by posting in a university showcase. After assessing the eligibility for enrollment, the members of the study team informed potential subjects about the trial and obtained their oral and written informed consent. The exclusion criteria included an allergy to PEA and a history of neurological, dermatological, and/or cardiovascular diseases. Patients with a history of pain disorders and patients taking chronic pain medication were also excluded. The intake of analgesics (including anticonvulsive and antidepressant drugs) during the study period was prohibited. The participants were informed that withdrawal from the study, at any time, was allowed without compromising their relationship with their healthcare provider.

### 2.3. Randomization and Blinding

The randomization scheme was generated using an online randomization tool (http://www.randomization.com, accessed on 12 August 2022). The preparation, randomization, and blinding of the verum and placebo substances were performed by the institutional pharmacy. They were prepared in boxes labeled with the study title, the subject ID, and the designation “Substance 1” and “Substance 2”. The participants and all staff involved in the study were blinded to the order of intake. An employee not participating in the study resolved the coding at the end of the study.

### 2.4. Study Medication

Each participant received 90 tablets, which were either 400 mg of PEA or an optically identical placebo manufactured in our institutional pharmacy. The study substance was provided by Innexus Nutraceuticals (Nijmegen, The Netherlands). Probands were instructed to take the tablets at 8 h intervals. The last intake was in the morning before the study visit. To increase therapy adherence, a reminder was given via mobile phone. During the study visit, the remaining tablets were counted and disposed of. Participants were interviewed about lost tablets and their adherence to the intake interval. After a wash-out period of 8 weeks, the participants received the second box with another 90 tablets.

### 2.5. Interventions and Course of Study

**Figure 1:**
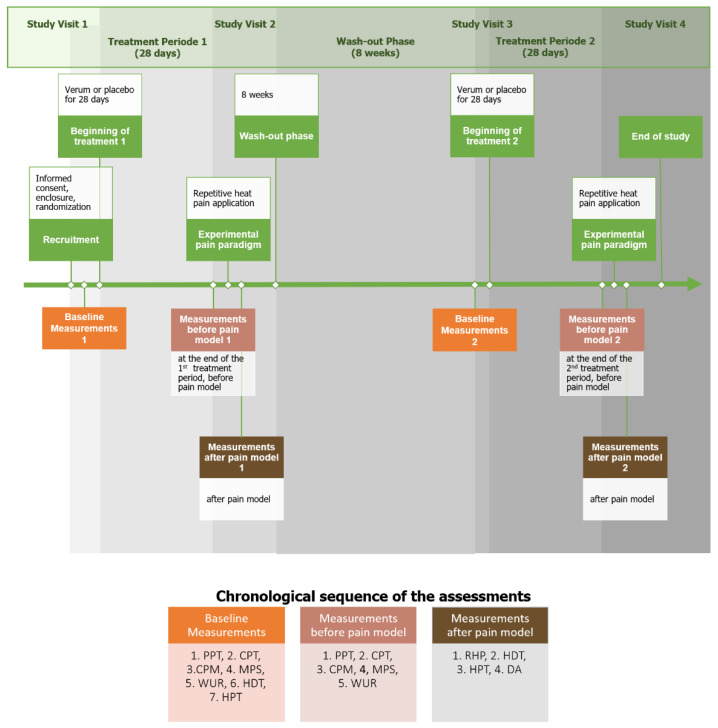
Schematic chart of the course of the study. The assessments were performed in the sequence presented in the chart. CPM: conditioned pain modulation, CPT: cold pain threshold, DA: distance of allodynia, HDT: heat detection threshold, HPT: heat pain threshold, MPS: mechanical pain sensitivity, PPT: pressure pain threshold, RHP: repetitive heat pain, WUR: wind-up ratio.

The course of the study is visualized in Figure 1.

**Figure 2:**
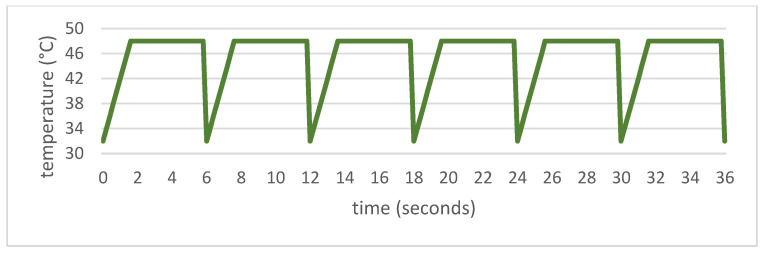
Time course of the thermode temperature in the “repetitive phasic heat application” pain paradigm. This block of six heatings was followed by a 30 s break. The whole procedure was repeated 10 times.

After enrollment, participants were randomly allocated into two interventions receiving either 400 mg of PEA or placebo three times daily. An initial baseline measurement was conducted. After 28 days of taking either the verum or the study drug, the effect on CPM, the wind-up ratio, and the mechanical pain sensitivity were measured. This was followed by the repetitive phasic heat application [20]. A 3 × 3 cm area on the middle of the volar forearm of the nondominant upper limb was repeatedly heated with a TSA-II Neuro Sensory Analyzer (Medoc Ltd. Advanced Medical Systems, Ramat Yishai, Israel). The thermode started at 32.0 °C, heated up to 48.0 °C at 10 °C/s, and cooled back to 32.0 °C after 6 s. This was repeated a total of six times (Figure 2). This was followed by a 30 s break. A total of 10 blocks with six repetitions each were performed.

At the end of the last block, the repetitive heat pain was determined using a visual analog scale (VAS). Since primary and secondary hyperalgesia are most pronounced at one hour after a repeated phasic heat application [20], the measurement—always proceeding in the same order—of the allodynia distance and the detection of mechanical pain sensitivity as well as the determination of the heat pain and detection thresholds were performed 60 min after the last heating session.

After a washout period of 8 weeks, a new baseline measurement was taken before participants crossed over to the other study intervention for 28 days. The same procedures and measurements were repeated as described above.

### 2.6. Measurements

At the initial visit, a questionnaire with demographic data was filled out. 

Conditioned pain modulation, the pain pressure threshold, and cold pain tolerance were assessed.

CPM is based on a “pain-inhibits-pain” concept that represents a mechanism of endogenous analgesia [22]. In the CPM paradigm, one noxious stimulus (the test stimulus), is applied in the presence of another noxious stimulus (the conditioning stimulus) that is transmitted elsewhere in the body [23]. An evaluation of pain modulation capabilities can serve as a step forward in individualizing pain medicine [24].

The test stimulus was applied with a pressure algometer (Wagner Pain Test Model FPK Algometer, Greenwich, CT, USA) at the M. adductor pollicis brevis of the nondominant hand. The probe area was 1 cm². The algometer was used to determine pressure pain thresholds. For this purpose, the pressure applied with the algometer was gradually increased by 0.5 kg/s until the participant felt a sensation of pain [25]. This pressure was defined as PPT1.

After PPT1 was determined, the participants were asked to immerse their dominant hand and wrist in cold water (4 °C) until the proband could no longer tolerate it. This time was taken as a parameter for cold pain tolerance (CPT). Immediately after removing the hand, the pressure pain threshold was measured again at the same site as before (PPT2) [22,26]. The CPM response is defined as a reduction in the perceived magnitude of pain from the test stimulus when it is delivered concurrently with the conditioning stimulus [27]. The amount of CPM response was calculated by this formula: (PPT2−PPT1)/PPT1 × 100.

#### 2.6.1. Wind-Up and Mechanical Pain Sensitivity

When testing temporal summation, the perceived pain intensity of a single pinprick stimulus was compared to a sequence of 10 pinprick stimuli of the same force repeated at a rate of 1/s (256 mN). A train of pinprick stimuli was given within a small area of 1 cm² approximately 5 cm proximal to the middle of the thermal stimulation area. The subject was asked to give a pain rating representing the pain at the end of the train using a numerical rating scale [28]. The wind-up ratio (WUR) was calculated as the mean pain rating of three trains divided by the mean pain rating for the single stimuli [25]. The response to the single pinprick stimulus was taken as the mechanical pain sensitivity (MPS).

#### 2.6.2. Repetitive Heat Pain

Immediately after the last thermal application, the repetitive heat pain (the pain generated by means of repetitive phasic heat application) was assessed by means of the VAS scale (0–100).

#### 2.6.3. Assessment of the Heat Pain and Heat Detection Thresholds

Before and after the thermal application, assessments of the thresholds of heat perception (the heat detection threshold) and heat pain sensitivity (the heat pain threshold) were carried out according to the guidelines of the German Research Network on Neuropathic Pain [25]. The skin was heated with a TSA-II Neuro Sensory Analyzer (Medoc Ltd. Advanced Medical Systems, Ramat Yishai, Israel). The proband was instructed to stop the heating via a stop button once he noticed the increase in temperature (heat detection threshold) or when he noticed a pulling, burning, or stinging sensation in addition to the feeling of warmth (heat pain threshold). Each measurement was performed three times, and the results were averaged [29].

#### 2.6.4. Detection of Allodynia

Starting from the center of the thermal application, a von Frey filament (128 mN) was applied to the skin in four radial lines at 5 mm intervals, first from the outside to the inside, followed by a measurement from the inside to the outside. The distance at which a sharp unpleasant sensation first occurred or ceased was determined. The eight measurements were averaged [30].

#### 2.6.5. Adverse Effects

The participants were questioned about possible side effects during each study visit.

### 2.7. Statistics

#### 2.7.1. Sample Size Calculation

The sample size was calculated on the basis of the data on repetitive heat pain published by Jurgens et al. in which repeated phasic heat application was tested in healthy volunteers [20]. The researchers reported a mean value of 72.2 for maximum pain with a standard deviation (SD) of 5.4. Using a highly conservative approach, we doubled the SD for our calculations (10.8). Based on these data, a sample size of 12 would have 80% power to detect a difference of 10 out of 100 VAS units in a t-test for paired samples with an alpha of 5%. In order to compensate for dropouts, our sample size was set to 14 participants.

#### 2.7.2. Statistical Analysis

The statistical evaluation was carried out with the Wilcoxon signed-rank test. Qualitative data were assessed with a chi-squared test. The effect size was calculated by dividing the z-score by the square root of N. The statistical analysis was performed with NCSS (Version 20.0.5, NCSS LCC, Kaysville, UT, USA).

## 3. Results

From February to May 2021, 14 subjects (8 women and 6 men) were included in this study. The participants’ characteristics are summarized in Table 1. All participants completed the study without deviating from the protocol. The results are shown in Table 2. 

### 3.1. Effect of PEA on Repetitive Heat Pain

Repetitive heat pain was significantly decreased after PEA intake. Patients reported VAS values of 41 ± 11 after PEA vs. 52 ± 13 after placebo at the end of the repetitive phasic heat application (*p* = 0.048).

### 3.2. Effect of PEA on CPT, PPT, and CPM

The assessment of the conditioned pain modulation paradigm resulted in three different variables. The cold pain tolerance was significantly prolonged at the end of the PEA treatment period (65 s ± 30 vs. 54 sec ± 27, *p* = 0.0023). The pressure pain tolerance was increased at the end of the PEA treatment period (7.7 kg ± 1.5 vs. 7.1 kg ± 1.5, *p* = 0.0013). The CPM was increased to 118% ± 12 vs. 110% ± 4 (*p* = 0.017).

### 3.3. Effect of PEA on Wind-Up Ratio

The wind-up ratio was significantly decreased at the end of the PEA treatment period (1.3 ± 0.3 vs. 1.5 ± 0.3, *p* = 0.043).

### 3.4. Effect of PEA on Allodynia (von Frey), MPS, HPT, and HDT

The average distance of allodynia was significantly decreased at the end of the PEA treatment period (1.6 cm ± 0.6 vs. 2.1 cm ± 0.8, *p* = 0.0011). The MPS was 23 ± 14 vs. 35 ± 14 on a 101-point VAS scale (*p* = 0.0092). The HDT was not significantly different between PEA and placebo (34.2 °C ± 2.3 vs. 34.05 °C ± 2.4, *p* = 0.97). The HPT was significantly higher at the end of the PEA treatment period (42.5 °C ± 1.4 vs. 41.4 °C ± 1.9, *p* = 0.014).

### 3.5. Pretreatment Baseline Data

To rule out a carry-over effect, baseline measures were conducted before both treatment periods. There were no significant differences between the two timepoints for all observed parameters (Table 3).

### 3.6. Adverse Events

No side effects or adverse events were reported by the participants in any intervention.

## 4. Discussion

Our data demonstrated the effect of PEA on acute pain in a human experimental model. The intensity of repetitive heat pain after the repeated phasic heat pain paradigm was reduced by more than 20% after PEA intake. This reduction is noteworthy in the context of the validation study of this paradigm, in which 800 mg of acetaminophen had no significant effect on repetitive heat pain [20]. However, it must be noted that in their study participants received a single dose after pain exposure, whereas in our present study, we applied long-term analgesia that started prior to the noxious stimulus. 

Our study did not only aim to prove the analgetic effect but also to give insights into the mechanisms involved. The chosen pain model induced two distinct types of physiological responses. First, due to the peripheral sensitization caused by an increased nociceptive input, an inflammatory process is activated in the course of an acute tissue trauma that causes a release of proinflammatory cytokines (IL-1, IL-2, IL-6, IL-7, and TNF), chemokines, and neutrophils. Consequently, primary hyperalgesia is developed after triggering the sensitization of peripheral nociceptors [31,32,33,34].

As a peroxisome proliferator-activated receptor-alpha ligand (PPAR-alpha), PEA reduces inflammation by inducing the expression of anti-inflammatory proteins and repressing the expression of proinflammatory cytokines, e.g., TNF-α. Furthermore, it acts by limiting the recruitment of immune cells [35]. These anti-inflammatory properties may counteract prosensitization cascades that drive peripheral sensitization [36]. By detecting the heat pain threshold, which is generally regarded as a marker of peripheral sensitization, we could clearly demonstrate the clinical relevance of the peripheral effect site of PEA [37]. In contrast, the heat detection threshold did not differ after the PEA or placebo treatments, which is possibly due to the fact that this parameter is less sensitive than the parameters defined as the heat pain threshold.

Another pivotal mechanism that was triggered by the pain model is central sensitization, referring to an amplification of pain represented by secondary hyperalgesia. Typical for this process are alterations in the mechanical sensitivity parameters such as mechanical pain sensitivity, pressure pain threshold, and the occurrence of allodynia [33,38]. It is also indicated that such an ongoing process alters the cold pain threshold [38]. The induction of central sensitization in humans is associated with the excitation of TRPV1-bearing primary afferents [39]. Furthermore, it is evident that microglia and astrocytes are also important in modulating synaptic plasticity, leading to central sensitization [40]. Both TRPV1 and glial pathways are influenced by PEA. Therefore, it could be shown that a normalization of formalin-induced microglial and glial activation occurred after PEA intake as well as an increased expression of glial interleukin 10 [41]. Ambrosino et al. detected TRPV1 activation and desensitization by PEA and suggested that it causes a higher degree of TRPV1 desensitization compared to capsaicin [42]. This mechanism may potentially contribute to the observed effect of PEA reducing central sensitization; however, our study was designed to elucidate clinical mechanisms but not on a cellular or molecular basis.

Furthermore, our study highlighted alterations in two dynamic somatosensory parameters. CPM is an experimental paradigm that captures endogenous pain modulation to examine the inhibitory mechanisms to a test stimulus (Ts) when a second noxious stimulus, i.e., the conditioning stimulus (CS), is concurrently applied [17]. It is generally regarded as a marker of the effectiveness of the inherent pain inhibition ability. It is well-documented that modulation of the noradrenergic transmission system affects this endogenous pain inhibition [43]. Wind-up describes the enhanced pain response to a series of stimuli compared to a single stimulus [17]. It is caused by the sensitization of dorsal horn neurons because of repetitive input from polymodal C-fiber nociceptors [44]. CPM and wind-up are mediated by a central mechanism. These two dynamic “quantitative sensory testing” (QST) measures can point to changes in central pain processing. 

Recently, the cannabinoid system has also received broader attention in this context, as it has been shown that the cannabinoid system is able to modulate the descending noradrenergic systems [45]. However, in the case of PEA, based on animal models it has been shown that the achieved peripheral antinociception is dependent on the noradrenergic system [46]. Furthermore, González-Hernández et al. suggested a reduction in the activity of the Aδ- and C-fibers while the nociceptive evoked responses of dorsal horn wide-dynamic-range neurons are inhibited [47].

Wind-up is mediated by repetitive inputs from polymodal C-fiber nociceptors, which are conducted primarily to the thalamus and subsequently to various domains of the brain [48]. Wide-dynamic-range dorsal horn neurons are implicated in this process. The involvement of the NMDA receptors in the development of wind-up was confirmed by the fact that NMDA receptor antagonists can inhibit wind-up [18].

No side effects were reported in our study population. In a small sample, of course, this is not very representative. However, in studies involving more than 1500 subjects, no adverse effects were found [49]. In a meta-analysis, PEA showed a high tolerability. The drop-out rate was significantly lower in the PEA group compared with the placebo group [7]. Overall, PEA is considered to be well-tolerated with a very low risk profile.

Taken together, our results suggest a central mechanism exerting ascending facilitation and descending inhibition of pain.

### 4.1. Limitations

The results of our study have been interpreted in the context of our study population. As we aimed to evaluate the underlying mechanism, the study design included a population with minimal confounders, which would always be present in the case of a clinical diagnosis. Therefore, we included healthy volunteers who had no concomitant medications or pain-related diagnoses. For this reason, assumptions for clinical settings should be drawn with caution.

One potential confounder could be caused by changes in pain perception during the menstrual cycle. We tried to minimize this risk by applying a 28 (or multiple) day rhythm between the measurements.

### 4.2. Clinical Application and Future Perspective

A combination therapy with different analgetic approaches, through which a desensitizing effect can be achieved by different mechanisms, is generally considered superior to monomechanistic therapy [49]. Our data provided the insight that PEA exerts its effect through multiple modes of action: reducing peripheral and central sensitization and increasing pain modulation. This explains its positive effect on various chronic pain conditions [7].

We assumed that the effect of PEA on central sensitization plays a pivotal role in alleviating chronic pain. Additionally, improved endogenous pain inhibition and decreased pain amplification also clinically contribute to analgesia. One can therefore deduct that PEA will be most effective in patients and diseases in which these aforementioned mechanisms are most important. Further trials could elucidate the efficacy in patients with reduced CPM, people suffering from depression, or in situations where central sensitization develops, such as fibromyalgia. Our data also suggest the effectiveness of PEA as a preventive analgesic treatment. This approach may be further explored in future trials, e.g., in the management and prevention of persistent postoperative pain.

## 5. Conclusions

Our present study has demonstrated that PEA has clinically relevant analgesic properties, acting on both peripheral and central mechanisms as well as in pain modulation.

## Figures and Tables

**Table 1:** Participants’ characteristics.

Sex (f/m)	8/6
Age (years)	40 ± 15
Weight (kg)	75.5 ± 11.6
Height (cm)	177 ± 11
BMI (kg/m²)	23.9 ± 2.1
Compliance of PEA intake	99.4%
Compliance of placebo intake	98.1%

BMI: body mass index.

**Table 2:** Results.

Parameter	PEA	Placebo	*p*	Effect Size
PPT (kg)	7.7 ± 1.5	7.1 ± 1.5	0.0013	0.86
CPT (s)	65 ± 30	54 ± 27	0.0023	0.82
CPM (%)	118 ± 12	110 ± 4	0.017	0.64
MPS (0–100)	23 ± 14	35 ± 14	0.0092	0.70
WUR	1.3 ± 0.3	1.5 ± 0.3	0.043	0.54
HPT (°C)	42.5 ± 1.4	41.4 ± 1.9	0.014	0.66
HDT (°C)	34.2 ± 2.3	34.05 ± 2.4	0.97	0.008
RHP	41 ± 11	52 ± 13	0.048	0.53
Distance of allodynia (cm)	1.6 ± 0.6	2.1 ± 0.8	0.0011	0.88

CPM: conditioned pain modulation, CPT: cold pain tolerance, HDT: heat detection threshold, HPT: heat pain tolerance, MPS: mechanical pain sensitivity, PEA: palmitoylethanolamide, PPT: pressure pain threshold, RHP: repetitive heat pain; WUR: wind-up ratio.

**Table 3:** Baseline Data.

Parameter	Baseline 1	Baseline 2	*p*	Effect Size
PPT (kg)	7.0 ± 1.5	7.0 ± 1.5	0.78	0.08
CPT (s)	54 ± 26	53 ± 24	0.31	0.27
CPM (%)	114 ± 9	112 ± 5	0.26	0.30
MPS (0–100)	40 ± 14	38 ± 14	0.51	0.18
WUR	1.5 ± 0.4	1.6 ± 0.4	0.22	0.33
HPT (°C)	44.4 ± 1.9	43.7 ± 1.6	0.23	0.32
HDT (°C)	34.6 ± 2.2	35.0 ± 2.3	0.47	0.19

CPM: conditioned pain modulation, CPT: cold pain tolerance, HDT: heat detection threshold, HPT: heat pain tolerance, MPS: mechanical pain sensitivity, PPT: pressure pain threshold, WUR: wind-up ratio.

## Data Availability

Data are available on request from the corresponding author.
